# Inorganic/organic combination: Inorganic particles/polymer composites for tissue engineering applications

**DOI:** 10.1016/j.bioactmat.2023.01.003

**Published:** 2023-01-11

**Authors:** Astha Sharma, Ganesh R. Kokil, Yan He, Baboucarr Lowe, Arwa Salam, Tariq A. Altalhi, Qingsong Ye, Tushar Kumeria

**Affiliations:** a School of Materials Science and Engineering, University of New South Wales, Kensington, Sydney, NSW, 2052, Australia; b Australian Centre for Nanomedicine, University of New South Wales, Kensington, Sydney, NSW, 2052, Australia; c Institute of Regenerative and Translational Medicine, Department of Stomatology, Tianyou Hospital, Wuhan University of Science and Technology, Wuhan, 430030, China; d Chemistry Department, College of Science, Taif University, Taif, 21944, Saudi Arabia; e Center of Regenerative Medicine, Department of Stomatology, Renmin Hospital of Wuhan University, Wuhan, 430060, China; f School of Pharmacy, University of Queensland, Woolloongabba, QLD, 4102, Australia

**Keywords:** Composites, Regenerative medicine, Inorganic nanomaterials, Mesoporous silica, Porous silicon

## Abstract

Biomaterials have ushered the field of tissue engineering and regeneration into a new era with the development of advanced composites. Among these, the composites of inorganic materials with organic polymers present unique structural and biochemical properties equivalent to naturally occurring hybrid systems such as bones, and thus are highly desired. The last decade has witnessed a steady increase in research on such systems with the focus being on mimicking the peculiar properties of inorganic/organic combination composites in nature. In this review, we discuss the recent progress on the use of inorganic particle/polymer composites for tissue engineering and regenerative medicine. We have elaborated the advantages of inorganic particle/polymer composites over their organic particle-based composite counterparts. As the inorganic particles play a crucial role in defining the features and regenerative capacity of such composites, the review puts a special emphasis on the various types of inorganic particles used in inorganic particle/polymer composites. The inorganic particles that are covered in this review are categorised into two broad types (1) solid (e.g*.,* calcium phosphate, hydroxyapatite, *etc.*) and (2) porous particles (e.g*.,* mesoporous silica, porous silicon *etc.*), which are elaborated in detail with recent examples. The review also covers other new types of inorganic material (e.g*.,* 2D inorganic materials, clays, *etc.*) based polymer composites for tissue engineering applications. Lastly, we provide our expert analysis and opinion of the field focusing on the limitations of the currently used inorganic/organic combination composites and the immense potential of new generation of composites that are in development.

## Highlights

• Inorganic/organic combination composites typically comprise of inorganic particulate material and a polymer matrix.
• Inorganic/organic composites mimic physiologic elements of natural tissue such as bones at cellular and subcellular levels.
• Solid and porous inorganic particles have been incorporated into polymers to form Inorganic/organic combination composites.
• Such composites display unique and enhanced chemical, electrical, and biological properties.

## Graphical abstract

**Figure:**
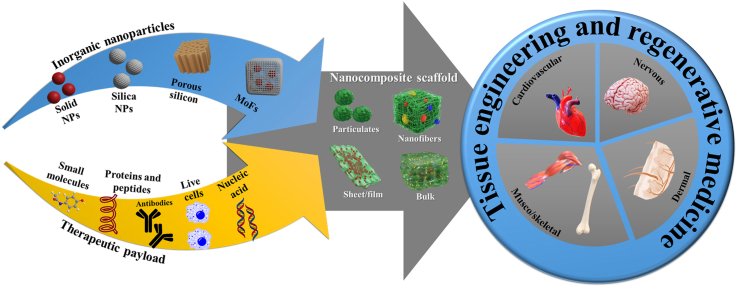

## 1 Introduction

Regeneration of injured tissue and restoration of body parts has been a long sought-after human desire dating back to ancient Egyptian civilizations [1]. Documented evidences reveal the unique material interventions employed for the post-mortem reunion and reconstruction of body parts, as it was thought to help the deceased achieve revitalisation and salvation in the afterlife [1]. Since then, the focus has shifted from reconstructing body parts for perceived benefits in the afterlife to addressing issues that come with living tissues in living humans. As such, the last two decades has seen an exponential rise in research efforts surrounding engineering biomaterials specifically tuned for the regeneration and restoration of various tissues. Much of these efforts have been concentrated on discovery of new ceramic, metallic, and polymeric biomaterials, while also evolving the preparation methods to generate materials with precisely designed regenerative potential [2]. This has given promise to the field of tissue engineering and regenerative medicine (TERM), a branch of medicine, and rise to its many sub-branches focusing on the regeneration of various tissues including cartilage bone [3] (e.g*.,* ear and joints) [4], skin [5], cornea [6], nervous system [7], cardiovascular [8] as well as dental [9] tissues, all of which either do not regenerate or have unacceptable or extremely slow rates of regeneration.

TERM is aimed at restoring a damaged tissue to their desired form and function, often involving the use of biomaterials. The biomaterials forms used in TERM are broadly categorised as scaffolds, grafts, and implants based on their roles. Metals, alloys, and ceramics are widely utilised in implants (both as load bearing and non-load bearing components) due to their pronounced mechanical properties and durability [10]. Whereas polymers and composites are materials chosen for scaffolds and grafts as a means of supporting damaged tissue and guiding healing in a controlled fashion [11]. The scaffold replicates the 3D matrix of the extracellular support system [12] of the defective organ and facilitates cell attachment, differentiation, and organization into a healthy new tissue [13]. A large part of the research efforts in TERM have been focused on soft polymeric scaffolds and grafts due to the wide range of available polymer chemistries, their ease of handling and processing, and the ability to incorporate therapeutic payloads for a localised therapeutic effect. The versatility and tunability of polymers, in addition to their biocompatibility, makes them an excellent material for TERM applications [14]. However, polymers are unable to mimic the multi-material composite environment of many natural tissues (e.g*.,* bone). Additionally, the requirement of harsh organic solvents for the manufacturing many popular polymer scaffolds (e.g*.,* polycaprolactone, poly-lactic-co-glycolic acid) makes them unsuitable for incorporation of water-soluble and sensitive macromolecular therapeutics (i.e*.,* proteins, peptides, antibodies, growth factors) [15]. Driven by these shortcomings, new composite materials incorporating various organic and inorganic materials in the polymer matrix gained exceptional popularity in the last few decades for TERM [16,17]. In this direction, polymer composites with carbon-based materials (e.g*.,* graphene, nanodiamonds, carbon nanotubes) [18], inorganic/ceramic particles (e.g*.,* silica, silicates, calcium phosphate, hydroxyapatite) [19] and metal or metal oxide particles (e.g*.,* silver, gold, iron oxide) [20] have been explored and employed for tissue engineering purposes.

**Fig. 1:**
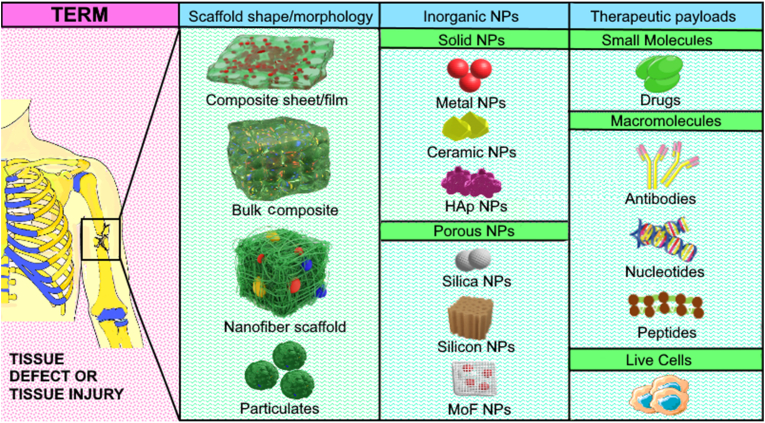
Schematic representation of use of various inorganic-organic polymer composite systems for use in tissue engineering and regenerative medicine (TERM). A tissue defect (left most: magenta-pink panel) in the form of a broken bone or connective soft tissue or skin wounds are fixed using various inorganic-organic polymer composites including films/sheets, nanofibers, particulates, and filling of defects with bulk composites as shown in the first green panel from the left under “Scaffold shape/morphology”. Commonly used inorganic materials can either be solid or porous and typically used ones are listed in the second green panel from the left under “Inorganic NPs” heading. The right most green panel shows the various types of drug payloads that can be incorporated into the inorganic nanoparticles for providing a localised therapeutic relief in combination with scaffold guided recovery. The particles are subsequently integrated with specifically designed material to create the desired nanocomposite scaffold for tissue engineering.

As depicted in Fig. 1, a polymer composites are typically a multi-phase system, in which particles (organic or inorganic) are covalently or physically incorporated into the polymer matrix with a specific purpose aimed at enhancing the mechanical, electrical, optical, and biological properties as required for the final product. For TERM, composites with enhanced biological properties and precisely tuned mechanical features are preferred to modulate regenerative outcome by means of protein adsorption, cell attachment, proliferation, migration, and differentiation [[21], [22], [23], [24]]. Several recent reviews have broadly covered the use of composites for tissue engineering applications [21,24]. However, only limited information is available on inorganic material and polymer matrix composites.

In this review, we comprehensively covered composites that incorporate various inorganic materials into a polymeric matrix. We detailed the advantages of composites over solely polymeric biomaterials for tissue engineering, followed by a brief outlook into the different types of composites covering organic particles/polymer composites and inorganic particles/polymer composites highlighting their advantages and disadvantages. Subsequently, the next sections provide a detailed account of various inorganic materials categorised into solid and porous materials for use in designing inorganic/organic combination composites for TERM. Lastly, we discuss the current challenges in the use of such composites and future opportunities.

## 2 Composites in tissue engineering

Despite the widespread use of scaffolds and grafts made from natural [25] or synthetic [26] polymers, ceramics [27], and bioactive glass [28], these materials on their own have proven to be inefficient for optimal tissue regeneration. Hence, multi-material composite biomaterials with the synergistic benefits of their various components are needed for tissue regeneration [29]. A multitude of composite combinations have been utilised for TERM that incorporate either a ceramic [30], metal [31], metal oxide [32], or organic materials [30] into an organic or inorganic matrix [33] aimed at fostering positive cell-biomaterials interactions, which are governed by the unique surface physio-chemical properties of the composite. Some of the surface properties which drive this interaction are surface charges, corresponding surface energies, and topography [34]. Chemical interactions between the various components of the composites are responsible for creating the overall properties of interest, particularly the mechanical and biological properties. In the following sections, we have characterised composites into two broad categories; (i) organic composites (comprised of organic particles in a polymer matrix); (ii) inorganic/organic composites (comprised of inorganic particles in a polymer matrix). With the focus being primarily on inorganic/organic combination composites and their applications in TERM, we specifically discuss advantages and limitations of each type of composite.

Before detailing the various composites, it is necessary to briefly cover the types of polymers that are used in TERM. Polymers are mainly categorised into two subtypes, natural and synthetic polymers. Natural polymers (e.g*.,* chitosan, collagen, hyaluronic acid) are ideal candidates for tissue engineering composites owing to their biocompatibility, high water retention capability, and degradability [[35], [36], [37]]. Despite these highly regarded qualities, it is difficult to design natural polymers that are personalised and have highly controllable rates of degradation. Alternatively, synthetic polymers like poly (glycolic acid) (PGA) poly(caprolactone) (PCL), and many others possess chemical, mechanical, and structural properties which are precisely tuneable and desirable for TERM. However, synthetic polymers may exhibit poor biocompatibility and the most widely used synthetic polymers release acidic by-products upon degradation, which may adversely affect the tissue micro-environment and regeneration process, whilst also affecting their mechanical properties [38].

### 2.1 Organic composites: organic particle/polymer system

As the name suggests, organic composites consist of only hydrocarbon-based organic components, typically generated by incorporating organic particles into a polymeric matrix. Organic composite materials exhibit characteristics such as biocompatibility, low toxicity, and biodegradability, and thus are extensively popular in tissue engineering applications [39,40]. A wide range of different organic particles have been successfully integrated into both natural and synthetic polymers to create composites with enhanced regenerative performances [41]. Carbon nanomaterials (e.g., carbon nanotubes, graphene, carbon nanofibers) [18] and polymeric particles have been used as additives for the generation of organic composites. Typically, the additives are selected based on the requirements of the final application. For e.g., carbon nanotubes, graphene, or filamentous nanomaterials have been added to achieve improved mechanical properties, localised release of therapeutics payloads, and especially electrical conduction [42]. Ginestra (2019) engineered a porous nanofibrous scaffold via electrospinning a solution of poly-(ε-caprolactone) (PCL) and various concentrations of graphene (0, 1 and 2 wt%). The concentration of graphene affected both the dimensions and homogeneity of the nanofibers. With an increase in graphene content, the nanofibers became thicker and presented higher variability in distribution (p-values of 0.210 for 2% of graphene compared to 0.020 for 0% of graphene). The presence of graphene also increased the elastic modulus from 5.6 ± 2 MPa for 0% to 21 ± 3 MPa and 22.5 ± 5 MPa for 1% and 2% of graphene embedded PCL nanofibers, respectively. The nanofibers were then tested for their ability to differentiate neural stem cells to dopaminergic neurons by analysing the presence of tyrosine hydroxylase after a culture period of 5 days. The dopaminergic neurons were present at a lower count and non-uniformly dispersed in the PCL nanofibers without graphene, as compared to PCL samples with graphene. Thus indicating that the presence of the graphene enhanced the differentiation of neural stem cells in dopaminergic neurons [43].

**Fig. 2:**
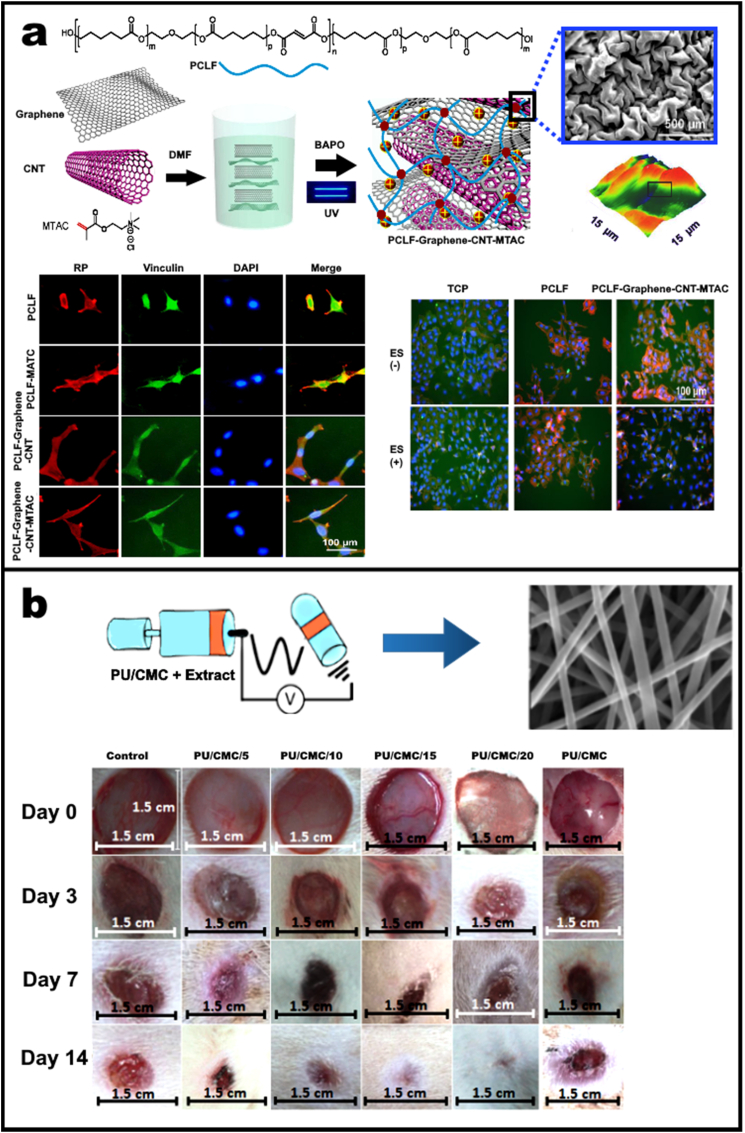
(a) Schematic representation of photo crosslinking-based fabrication of PCLF–Graphene–CNT–MTAC scaffolds. SEM and AFM of the scaffolds confirms the rough surface possessing tubular and sheet-like structures suitable of cell adhesion. Immunofluorescence staining was performed for nerve growth factor-induced differentiation of PC12 nerve cells after 3 days on PCLF, PCLF-MTAC, PCLF–Graphene–CNT, and PCLF–Graphene–CNT–MTAC scaffolds presenting cellular F-actin (red), vinculin (green), and nuclei (blue). PCLF–Graphene–CNT–MTAC scaffolds showed improved neurite extension and nuclear elongation. Effect of ES on PC12 cells cultured on PCLF–Graphene–CNT–MTAC sheets and control TCP and PCLF substrates. ES was provided at 100 mV/mm−1 and 20 Hz for 2 h per day for a total of 7 days. Cellular growth was also found to be amplified for scaffold when compared to control or substrate after exposure to ES. Reproduced from Ref. [44]. (b) PU-based nanofibers wound dressings containing Malva sylvestris with different amounts of CMC. PU/CMC nanofibers showed no antibacterial activity against *S. aureus*, and *E. coli*. The antibacterial increased with increase in herbal extract against *S. aureus* and *E. coli*. Wound healing on an animal in each group on days zero, three, seven and 14 after treatment showed better healing than gauze covered wounds which can be due to the higher fluid absorption value of PU/CMC dressing compared to gauze bandage (320.5%) and bacteria barrier property. Reproduced from Ref. [47].

In another study, Sun et al. (2020) fabricated an electrically conductive and positively charged scaffold by incorporating graphene, carbon-nanotubes, [2-(methacryloyloxy)ethyl] tri-methylammonium chloride in poly(caprolactone-fumarate) (PCLF–Graphene–CNT–MTAC) through photo crosslinking. As shown in Fig. 2a, SEM image of PCLF–Graphene–CNT–MTAC scaffolds presents rough surface with tubular and sheet-like structures. AFM profiles of PCLF–Graphene–CNT–MTAC also confirmed surface complexity at a biological scale appropriate for cellular adhesion. The neural differentiation of PC12 nerve cells after 3 days was studied via immunofluorescence staining of cells on PCLF, PCLF-MTAC, PCLF–Graphene–CNT, and PCLF–Graphene–CNT–MTAC scaffolds. As represented in Fig. 2a, PC12 cells on the PCLF–Graphene–CNT–MTAC scaffold showed elongated nuclei more than cells on the other scaffolds. The PCLF–Graphene–CNT–MTAC scaffold was further investigated to study the effect of ES (electrical stimulation) on PC12 cells. With ES, PCLF–Graphene–CNT–MTAC presented significantly more cell spreading and neurite extension after 7 days, though no significant difference was observed on either the control TCP (tissue culture polystyrene) or PCLF substrates. Thus, the scaffold demonstrated enhanced surface charges, surface roughness, and electrical conductivity, leading to improved biocompatibility and the promotion of PC12 cell attachment and proliferation [44].

A common issue with number of carbon nanomaterials is their hydrophobic nature that requires the use of organic solvents during the production of composite scaffolds. This hinders the incorporation of protein payloads and prevents the use of aqueous soluble polymers as the matrix, hence limiting their applicability [45].

On the other hand, polymeric particulates enable delivery of a therapeutic payload from the scaffold [46]. For example, polyurethane (PU) based nanofibers were integrated into a carboxymethyl cellulose (CMC) matrix to elute a herbal antidiabetic, *malva sylvestris*. These composite nanofibers presented the dual benefits of being an anti-inflammatory and antimicrobial dressing, intended for use in a diabetic wound-healing application as represented in Fig. 2b [47]. Here, varying amounts of CMC and PU were tested for their absorption ability of wound exudates. It was observed that 20% w/w CMC in the polymer blend led to the steady release of the herbal extract. *In-vitro* investigation showed increased macrophage infiltration, neovascularization activity, and fibroblastic proliferation in this composite scaffold on the 7th day, post-incubation. Furthermore, by the 14th day the extent of collagenization and epithelium regeneration was boosted by these scaffolds. Overall, organic components offer a great range of matrices with tuneable micro- and macroscopic structural features with number of flexible processing protocols. Additional examples are summarised in Table 1.

**Table 1:** *Examples* of various categories of composites along with their application.

System	Materials	Function	References
Organic Composites (Organic particles/organic polymer matrix)	Poly(3-hydroxybutyrate), reduced graphene oxide and polyaniline	Bone regeneration by stimulating bone callus formation	[143]
	Poly l-lactic acid and cyclic olefinic copolymer	Bone Tissue Engineering.	[144]
	Connective tissue growth factor encapsulated poly-lactic acid- polyvinyl alcohol (PLA-PVA) core-shell fiber	Type 2 diabetic wound healing	[145]
	6-deoxy-6-hydrazide Cellulose (Cell Hyd) 6-deoxy-6-diethylamide Cellulose (Cell DEA) and 6-deoxy-6-diethyltriamine cellulose (Cell DETA)	Tissue engineering applications	[146]
Inorganic/organic combination: Inorganic particle/polymer system	Strontium-hydroxyapatite and PCL	Improved wetting behaviour for tissue healing	[147]
	Calcium phosphate and whey protein isolate (WPI) gelatin	Bone tissue engineering and regenerative	[148]
	Hydroxyapatite, and collagen-carboxymethyl cellulose	Bone tissue engineering	[149]
	Bioactive nanohydroxyapatite particles, and poly-l-lactide	Medical Implants	[150]
	Zirconium, and PCL	Bone regeneration	[151]
	Zinc, and polyurethane–gelatin (PUG)	3D bioprinting	[152]
	Copper, and polydopamine	Prevents stent thrombosis and restenosis	[104]
	Strontium, and 1,3,5 tricarboxylicbenzene (H3BTC)	Orthopaedic applications	[153]
	Copper, and benzene-1,3,5-tricarboxylate (BTC)	Vascular tissue response	[154]
	Copper and 5-methylisophthalic acid (H_2_mica) and 1,3-bis(5,6-dimethylbenzimidazol-1-yl) propane (L)	Inhibits over-activity of dopaminergic neurons	[155]
	pSi infiltrated, and NGF payload	Treatment of neurodegenerative diseases	[156]
	pSi infiltrated, and poly(lactic-co-glycolic acid)	Enhance neuronal growth	[90]
	pSi polymeric replica, and polystyrene	Biosensing	[157]
	pSi polymeric replica, and polyurethane	Bone tissue engineering	[158]
	Polymer capped pSi, and poly[ethylene glycol-block-(dimethylaminoethyl methacrylate-co-butyl methacrylate)]	microRNA inhibitory peptide nucleic acids	[159]
	Polymer capped pSi and polyethyleneimine	Delivery of siRNA	[160]
	Polymer coated pSi, and poly 2-(diethylamino)ethyl methacrylate	Thermo-responsive anti-bacterial for wound dressing	[161]
	Polymer coated pSi, and polyetheretherketone	Anti-bacterial implant	[162]
	pSi film supported by poly(caprolactone)	Potential bone graft	[163]
	pSi film supported by poly(1,7-octadiene) and poly(acrylic acid)	Non-invasive decontamination of wounds	[164]
	pSi particles encapsulated by poly(lactide-co-glycolide), poly-l-lactic acid, PCL	DNA-based responsive devices	[165]
	pSi particles encapsulated by poly(vinyl alcohol)	Guided tissue regeneration	[84]
	pSi particles encapsulated by PCL	Guided cell growth, photoluminescence, and release bioactive proteins	[83]
	pSi particles encapsulated by PLGA	Neural growth	[90]

### 2.2 Inorganic/organic combination: Inorganic particle/polymer system

Inorganic/organic combination composites are typically made by incorporating inorganic materials (as particulates) into an organic polymer matrix. This combines the favourable properties of inorganic materials with ease of processability of the polymers [48,49]. Generally, the organic polymer matrix acts as a structural backbone that is designed to mimic the microscopic features of extracellular matrix (ECM). The ECM plays a crucial role in facilitating cell growth, attachment, and even has a part to play in guiding cellular differentiation. The inorganic components either supplement the overall composite by imparting unique properties such as high electrical and thermal conductivity, photoluminescence, bactericidal effect, or conversely, they enhance the pre-existing mechanical or structural features of the matrix. For e.g., Nazari et al. (2019) developed an ECM mimicking nanofibrous scaffold with electrically conducting properties to regenerate functional cardiac tissue [50]. Their ECM was composed of polymer combined with molybdenum disulphide (MoS_2_) nanosheets that imparted electrical conductivity to the scaffold as well as improving its mechanical strength. Furthermore, incorporation of MoS_2_ nanosheets induced cardiogenic differentiation and maturation of embryonal carcinoma cells (mECCs) without the need for cardiogenic biochemical supplements (i.e*.,* anabolic growth factors). This unique electrically conducting composite was found to be suitable as a microenvironment regulator for cardiac cells regeneration [50].

In another study, gellan gum (organic) was combined with TiO_2_ nanotubes (inorganic) and transformed into a transparent film by means of the solvent cast method. This resulted in enhanced cell proliferation, making the fabricated composite a suitable candidate for skin tissue engineering applications [51]. Both natural and synthetic polymers have been widely used for creating many novel inorganic/organic combination composites. The use of these composites in tissue engineering has continued to expand with the development of new inorganic materials. Similarly, the organic composites polymers in the inorganic/organic composites provide a structural backbone while the inorganic material additives enhance the intended functional qualities of the composite. To expand on this, the next few sections are dedicated to covering a range of inorganic materials that have been incorporated into polymers to form inorganic/organic combination composites along with relevant examples and an extended examples list detailed in Table 1.

## 3 Solid inorganic particle-based polymer composites for TERM

Solid inorganic particles, made from a range of different materials in varying shapes and sizes [52] are popular additives for composites used in TERM. Solid inorganic particles enhance the mechanical, biological, and electrical properties of scaffolds as a function of their unique chemical and structural features [53]. Solid inorganic particles are synthesised either using a bottom-up or top-down synthesis approach [54]. The top-down approach operates by breaking up the bulk material into smaller particles [55]. Conversely, the bottom-up approach involves the assembly of atoms and molecules to form particles in a medium. Some of the methods in this category include techniques like hydrolysis, chemical vapor deposition, micro-emulsion, chemical synthesis and thermal decomposition [56]. Advances in particle production and control over nanoscale organizations have given new direction to the synthesis (and in turn, the application) of composites in TERM. The most widely used solid inorganic materials for TERM are gold, silver, calcium phosphate, and hydroxyapatite. In the next section, composites made using these particles are discussed in detail with a spotlight shone on few recently explored examples.

### 3.1 Gold particle-based polymer composite

**Fig. 3:**
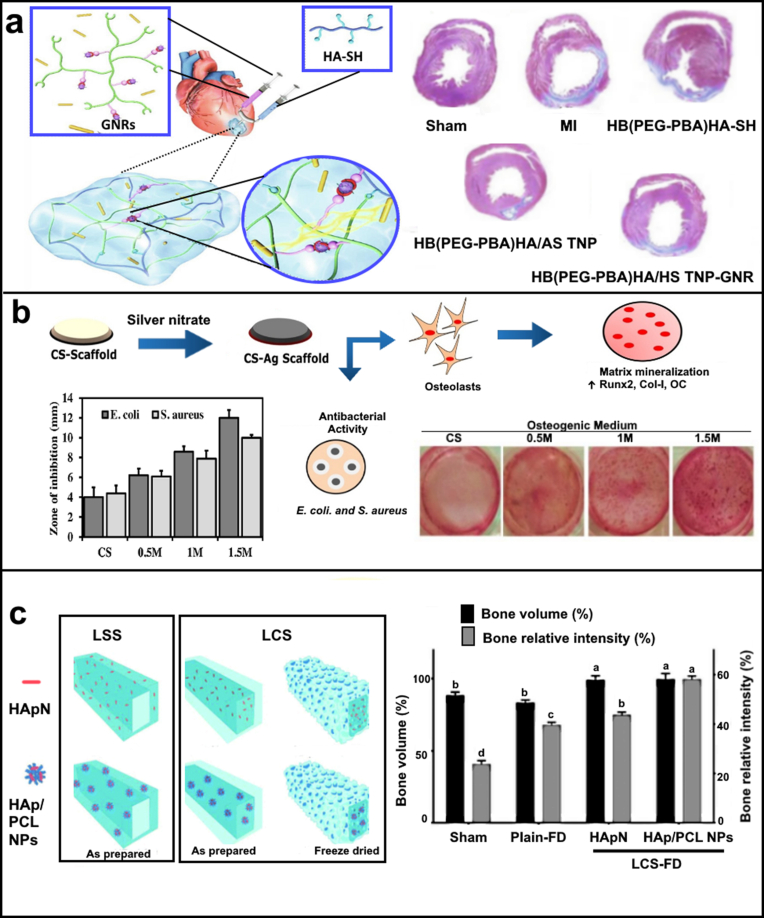
(a) Depicts an injectable hydrogel hybrid for cardiac regeneration comprising of phenylboronic acid hyperbranched polymers and thiol hyaluronic acid for loading Astragaloside IV and gold nanorods. The treatments of hydrogels significantly resisted these pathological and morphological changes, with the highest improvement by HB (PEG-PBA)/HA-SH/AST NPs/GNRs. Reproduced from Ref. [59]. (b) Composite scaffolds fabricated utilising CS and AgNPs. Graph shows that CS-Ag scaffolds exhibited greater antibacterial activity compared to naïve CS scaffolds against both *E. coli* and *S. aureus*. CS itself possesses antibacterial activity and it is greatly enhanced by the presence of silver nanoparticles. Alizarin red stained photographic images showed that cells grown on CS-Ag-1 M and CS-Ag 1.5 M scaffold films showed more prominent nodules compared to cells grown on other scaffold films. Reproduced from Ref. [67]. (c) Two different designs were realised for loaded-core scaffolds (LCS) and loaded-shell scaffolds (LSS), where PPI-4 was used to print plain shell and core phases for LCS and LSS, respectively, while HAp-ink and HAp/PCL NP ink were separately used to print core and shell phases in LCS and LSS, respectively. Immense tissue cavitation could be recognised in deeper layers as radiolucent regions among areas of higher radiopacity, greatly according with the significantly (p ≤ 0.05) lower percentage bone relative intensity recorded for the sham group compared to the Plain-FD group. Reproduced from Ref. [68].

Nanosized gold particles (AuNPs) are one of the most widely explored multimodal nanoparticles because of their unique plasmonic and chemical properties. AuNPs are shown to be highly tuneable to numerous shapes (spheres, rods, pyramids, and many others) and sizes, achieved by simple alterations in synthesis processes [57]. Nasir et al. (2017) synthesised amine functionalised AuNPs to conjugate to porcine derived cholecystic extracellular matrix (ECM) scaffold. The modified scaffold displayed no toxicity and supported the growth and proliferation of H9c2 cells (cardiomyoblasts), making it a potential biomaterial candidate for cardiac tissue engineering [58]. Chen et al. (2021) reported hyaluronic acid (HA) based injectable hydrogel, laden with Astragaloside IV (AST) nanoparticles or gold nanorods (GNRs) exhibited enhanced electrical conductivity. Thus, the injectable hydrogel improved myocardial infarction (MI) induced cardiac dysfunction and cardiac restoration by stimulating angiogenesis, inhibiting cell apoptosis and, promoting cell–cell signal transduction (Fig. 3a) [59]. In another study, Liao et al. (2021), fabricated multi-material composite that incorporated gold nanorods and bifunctional nanohydroxyapatite (nHA) in a methacrylated gelatin/methacrylated chondroitin sulphate hydrogel for photothermal bone tumour therapy and bone regeneration. The hybrid hydrogel showed dual functionality of tumour therapy and bone regeneration, thus demonstrating a new hope for tumour-related complex bone issues [60].

The integration of AuNPs within the scaffold can also be achieved by synthesizing the NPs *in situ* [61]. Lee et al. (2018) demonstrated a technique where gold NPs (AuNPs) were grown onto a 3D printed polycaprolactone (PCL) substrate coated with polydopamine (PDA) [62]. The PDA coating on the PCL substrate functioned as a reducing agent to enable a homogeneous growth of AuNPs onto the final scaffold. They confirmed that growth of AuNP was due to reducing nature of PDA as no AuNPs deposited on the uncoated PCL scaffolds. The scaffold with AuNP induced bone differentiation both *in vitro* and *in vivo*. Leaching of AuNPs and gold ions is a concern which should be carefully considered before any use in TERM or biomedical applications, which is correctly pointed out in this study and others. Therefore, detailed investigations of the long-term side-effects of AuNPs and Au ions is required before any tissue engineering scaffolds involving AuNPs is approved for clinical use.

### 3.2 Silver particle-based polymer composite

Silver (Ag) is known for its broad-spectrum as an antibacterial agent [63]. This antimicrobial activity is specific to its ionic form (Ag^+^), as it can disrupt the bacterial cell membrane inhibiting ATP production and subsequently constraining enzymatic activity and DNA replication [64]. Since prevention of bacterial infection and invasion is of utmost importance following a dental or orthopaedic graft surgery, scaffolds and substrate incorporating Ag have become wildly popular due to their broad spectrum antibiotic action [65].

In similar direction, Srivastava et al. (2019) reported the use of AgNPs to generate a silk fibroin mat with bactericidal properties for skin tissue regeneration and wound healing applications. The first stage of this process involved the electrospinning of an ionic solution of tasar silk fibroin to form nanofiber linings (mat), which were functionalised with dandelion leaf extract (*Tridax procumbens*) to enable *in situ* AgNPs generation. The addition of the AgNPs increased both the mechanical strength and water retention capacity of the matrix. The incorporation of AgNPs not only imparted antibacterial benefits, but also supported the proliferation and differentiation of fibroblast cells [66]. Besides its applicability to antibacterial applications, the role of AgNPs for cell attachment and proliferation promotion has also been investigated. In this direction, Vaidhyanathan et al. recently (2021) synthesised an AgNPs-based biodegradable chitosan composites for tissue engineering, utilising inherent reducing nature of chitosan. This enabled *in situ* AgNPs generation without the need of an external reducing agent. The composite scaffolds effectively supported osteoblast growth as well as osteogenic differentiation by the up-regulation of osteogenic markers and mineralization of the matrix, making it a promising biomaterial for bone tissue engineering as represented in Fig. 3b [67].

### 3.3 Calcium phosphate particle-based polymer composite

Calcium phosphate (CaP) as a biomaterial is of special interest for the scientific community focused on bone regeneration due to its chemical and crystallographic similarities to the inorganic components of native bone [69]. Because of these reasons, CaP is one of the few inorganic biomaterials that are approved for use in clinic with multiple products in the market (over-the-counter supplements, antacids, toothpaste, bone graft substitutes *etc*). It is known that incorporation of CaP particles into polymeric scaffolds enriches the matrix protein adsorption profile of the scaffold surface, which ultimately encourages better cell adhesion and drives desired cellular phenotypic differentiation [[70], [71], [72]]. The frequently applied forms of CaP are monocalcium phosphate anhydrous, monocalcium phosphate monohydrate, dicalcium phosphate anhydrous, dicalcium phosphate dihydrate, octacalcium phosphate, α- and β-tricalcium phosphate (TCP) [73]. Biphasic calcium phosphate (BCP) is one of the promising inorganic materials that is regularly used in combination with polymers to form composite tissue engineering scaffolds [74]. BCP is a mixture of hydroxyapatite (Ca_10_(PO_4_)_6_(OH)_2_), and β-tricalcium phosphate (Ca_3_(PO_4_)_2_). The incorporation of biphasic calcium phosphate particles (BCP NPs) into chitosan/gelatin hydrogel has shown promise for regeneration of bone defects. The bone marrow mesenchymal stem cells (BMSCs) cultured onto this composite demonstrated enhanced cell proliferation and induced their differentiation into osteoblast phenotypes. The implantation of the hydrogel into a rabbit femoral defect, revealed new bone formation in the scaffold core, which gradually increased over time. Hematoxylin and Eosin (H&E) staining revealed new bone regeneration at 1st month and by 3rd month blood vessels were formed, facilitating formation of new bone [75]. In addition, therapeutic payloads can be loaded onto nanostructured CaP to be released gradually at the site of injury over an extended period. Chen et al. (2017) demonstrated the effect of this strategy in a collagen-based composite containing dexamethasone (DEX) loaded BCP NPs. This work harnessed the slow and localised release of DEX to drive the osteogenic differentiation of BMSCs to promote ectopic bone formation in athymic nude mice [76].

### 3.4 Hydroxyapatite particle-based polymer composite

In the last 15 years, hydroxyapatite (HAp) has received a tremendous amount of attention as a notable biomaterial especially in the realm of TERM [77]. HAp has been extensively used for numerous medical applications as a scaffold material in prosthesis revision surgery, metallic implant coating, artificial and drug eluting bone grafts, and bone fillers [78]. A recent study by Rezk et al. (2020) showed composite nanofibers consisting of poly(glycerol sebacate) (PGS) and PCL laden together with a mixture of simvastatin (SIM) and HAp to mimic bone ECM, thereby enhancing bone cell adhesion, proliferation, and biomineralization [79]. Loading and delivery of bone morphogenetic protein-2 (BMP-2) from a mesoporous HAp-based silk fibroin/chitosan composite scaffold was demonstrated by Qui et al. (2020). The HAp nanoparticles (NPs) were designed to integrate BMP-2 into the scaffold to enable a controlled, sustained release of a protein-based therapeutic at the site of injury. The nanocomposites scaffold supported the growth of BMSCs, whilst also inducing their osteogenic differentiation and formation of bone tissue *in vivo* [80].

Methods of integrating HAp NPs into the composites differ as a function of the constituent material properties, application of the final product, and its cost. Yu et al. (2017) applied microwave-hydrothermal method to synthesise copper (Cu)-doped mesoporous HAp microspheres (Cu-MHMs), which were subsequently combined with chitosan to form a biomimetic scaffold (Cu-MHM/CS). When implanted into critical-sized calvarial defects in rats, the Cu-MHM/CS scaffolds significantly enhanced bone regeneration accompanied by new blood vessel formation at 8 weeks post-operation compared to the MHM/CS scaffolds. These results suggested that Cu-MHM/CS scaffolds could encourage bone regeneration by enhancing osteogenesis and angiogenesis simultaneously [81].

Using another method, El-Habashy et al. (2021) engineered core-shell structured osteoconductive hydrogel scaffolds using extrusion 3D printing of bio-inks incorporating bioactive hydroxyapatite/polycaprolactone nanoparticles (HAp/PCL NPs) (Fig. 3c). In their core-shell scaffold, the core was reinforced with HAp/PCL NPs and the scaffolds were freeze-dried (HAp/PCL NPs-LCS-FD). The scaffolds showed optimum controlled swelling behaviour whilst maintaining the structural integrity for 28 days. Both, cellular and in-*vivo* studies suggest the scaffolds with bioactive core offer a superior osteogenic and osteoconductive environment. *In-vivo* bone regeneration assessments in a tibial bone defect model in New-Zealand rabbits demonstrated that freeze-dried HAp/PCL NPs-LCS-FD scaffold led to near complete bone defect regeneration at six-week point compared to relevant controls as measured by computed tomography (Fig. 3c) [68]. All the afore mentioned studies and the ones summarised in Table 1 demonstrate that HAp-based composite scaffolds are highly conducive to for bone regeneration.

## 4 Porous particle/polymer composites for TERM

Material-cell interaction is an important aspect of regeneration, which continues to be at the forefront of research regarding guided cell growth and the development of novel composite materials. Composites that incorporate porous particles opens the whole new range of possible unique material-cell interactions. Moreover, with their high surface areas, open pore volumes, and porosities, the porous particles in the composite scaffolds enable features like incorporation and delivery of sensitive therapeutic payloads [82]. In addition, the porosity of the particles at the nanoscale can readily alter the biochemical reactivity of the material to achieve desired bioactivity or biodegradability of the final product. Some common examples of inorganic particles used in composites are porous silicon, mesoporous silica, and metal organic frameworks (MoF). These porous materials have been used with a variety of polymers to form composites for various TERM applications. The following sections aim to define the important features of these porous particles and elaborate on some of the recent examples of composites that utilize these materials for tissue regeneration.

### 4.1 Porous silicon/polymer composites

**Fig. 4:**
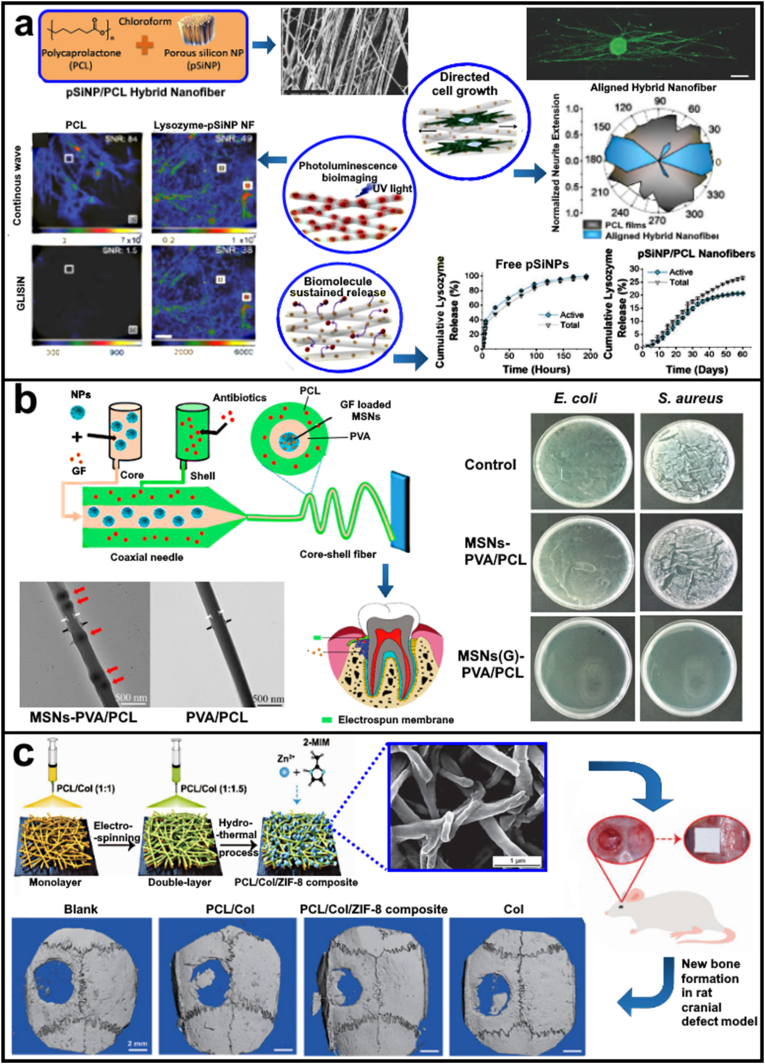
(a) Spray nebulization is used to produce nanofibers of polycaprolactone embedded with porous silicon nanoparticles (pSiNPs). Orientation analysis of astrocytes cultured on aligned hybrid nanofibers showed significantly greater alignment, with an average angle from the median angle of alignment of 6 ± 8°. Luminescence microscope images of control PCL fibers and lyso-pSiNP/PCL hybrid nanofibers obtained under steady state imaging conditions (top) and with time-gating (bottom). Time gating removes the prompt emission and scattered light from the image. Because pure PCL has no long-lived luminescence, the GLISiN image is black. Signal-to-noise ratios (SNR) are given for the regions of interest (ROIs) indicated with the white box in each of the images. Reproduced from Ref. [83] (b) Schematic Illustration of the Overall Process of Multifunctional Electrospun MSNs-Encapsulated Core–Shell Nanofibers with Growth Factor and Antibiotic Delivery Ability for GTR. Inner structure of the nanofibers as reveled by TEM showed a peapod like structure. Gentamicin-loaded MSNs–PVA/PCL membranes demonstrated a superior bactericidal effect against both types of bacteria. Compared to the control groups, MSNs–PVA/PCL nanofibers with gentamicin showed a reduction of 7 orders of magnitude in the CFU count (0 vs 107 CFU/mL). Reproduced from Ref. [84] (c) The asymmetric double-layer membrane was fabricated using a polymer blend of PCL and Col (1:1 and 1:1.5) based on the monolayer by electrospinning. The ZIF-8 crystals were subsequently formed *in situ* on one side of the double-layer membrane using a hydrothermal strategy. 3D micro-CT reconstructions of the defects after 8 weeks post-surgery in different groups. 3D reconstructed images displayed that bone-healing efficacy followed the following progression: Blank < PCL/Col < Col < PCL/Col/ZIF-8 composite group. Reproduced from Ref. [85].

Porous silicon (pSi) is a nanostructured silicon that has been widely explored for its applications in optics, microelectronics, and chemical/biological sensors owning to their semiconducting properties and facile fabrication processes for large scale production [86]. The pSi possesses properties such as high porosity, controllable pore dimensions, high loading capacity, tuneable surface chemistry, biodegradability, and biocompatibility. These properties make pSi an attractive inorganic material additive for composite scaffolds as particle dimensions, morphology, and porosity of pSi materials can be tuned according to the needs of the injury site [87]. The pSi nanoparticles (pSiNPs) can be readily combined with polymers to create composites with unique chemical, optical, and biological properties [88,89]. For example, oriented composite nanofibers containing pSiNPs embedded in a polycaprolactone or poly(lactide-co-glycolide) matrix directed growth of single rat dorsal root ganglion (DRG) cells and Neuro-2a. The pSiNPs in the composite nanofibers allowed localised delivery of sensitive biological therapeutic payloads, which are otherwise incompatible with PCL due to the need of harsh organic solvents during fabrication. Moreover, the study shows a sustained release of lysozyme (with >75% bioactivity retention) from the composite nanofibers for 60 days. The study also proposed the use of the inherent photoluminescence (PL) pSiNPs for scaffold health monitoring. As shown in Fig. 4a, the work reported that the ultra-long PL emission lifetime of pSiNPs allowed the suppression of the shorter-lived autofluorescence signal from living cells. The time-gated imaging method commonly known as gated luminescence imaging of silicon nanoparticles (GLISiN), achieved via acquisition of the emission image at a delayed time point from the pulsed excitation, such that the autofluorescence from cells or tissues is not detected. Here, GLISiN images pSiNP/PCL fibers displayed signal-to-noise ratio (SNR) between 20 and 80, whereas the SNR in GLISiN images of control PCL fibers was <2. Thus, the GLISiN images showed a 40-fold improvement in image contrast. The inherent PL of the pSiNPs in the nanofibers allow for PL based monitoring of the scaffold Fig. 4a [83].

In another study, Zuidema et al. (2020) incorporated therapeutic cargo loaded pSiNPs into the poly(lactic-co-glycolic acid) (PLGA) nanofiber scaffolds that permit the slow release of therapeutic agents to improve nerve injury reparation after traumatic episode. The work demonstrates the versatility of pSiNPs as a carrier by incorporating three different types of therapeutic molecules: 1. Nucleic acid (tropomyosin-related kinase receptor type B (TrkB) aptamer), 2. Small molecule drug bisperoxovanadium (HOpic) (bpV(HOpic)), and 3. Protein (nerve growth factor (NGF)). The pSiNPs loaded with these payloads were embedded into PLGA nanofiber scaffolds made using nebulization based method. Release kinetics of each payload was studied in-vitro. The drug-loaded pSiNP-nanofiber hybrids released approximately half of their TrkB aptamer, bpV(HOpic), and NGF payload in 2, 10, and >40 days, respectively. PLGA fibres itself induced lengthier neurite extension from DRG explants but the addition of bpV(HOpic)-pSiNPs, TrkB aptamer-pSiNPS, and NGF-pSiNPs further expanded the length of neurite intersection approximately by 150%, 183%, and 183% respectively. Cellular migration out of the DRG explants was also quantified to evaluate the effect of hybrid scaffolds onto the growth of Schwann cells and fibroblasts. PLGA fibers themselves induced extensive cellular migration from DRG. While the addition of TrkB aptamer-pSiNPS or NGF-pSiNPs, the distance of cellular migration was significantly enhanced. Overall, the therapeutic payload incorporated pSiNPs-based nanofiber scaffolds increased neurite extension and cell migration relative to the drug-free control nanofibers [90].

The research on pSi nanoparticles is still emerging and the existing status on the research already demonstrates the potential of pSi to be used as platforms for tissue engineering. Despite this, the growing complexity of the pSi nano-systems requires a thorough assessment of the probable toxicity issues and in-depth knowledge of the mechanisms of nanoparticle–cell interactions, as well as the effects on the *in vivo* systems [91].

### 4.2 Mesoporous silica/polymer composites

Mesoporous silica materials (MSMs) are inorganic mesoporous nanoparticles produced by a simple bottom-up nano-synthesis approach. In this approach, desired pore structure/morphology is generated by self-assembly of surfactant molecules, which is used as templates for deposition of silica shell layer. The self-assembled surfactant template is selectively removed to generate pores that mimic the order and geometry of the template. This simple and highly versatile synthesis approach opens numerous opportunities for the use of MSMs in various industrial and biomedical application [92]. MSMs have become highly popular nanocarrier materials for biological and pharmaceutical applications due to their highly tuneable structure, pore features, surface chemistry, ultrahigh surface area, and proven biocompatibility. These features also make MSNs attractive as additive in polymeric composites for various tissue engineering applications. Thus, many studies have explored their use primarily as a carrier to enable controlled release of therapeutic payloads from scaffolds and as additives to modify the mechanical and chemical features of the composite material. In a study by Kaliaraj et al. (2017), coated an inorganic mesoporous material, SBA-15 silica, with organic PLGA *via* micro-emulsion technique to generate bone bio-scaffold. *In vitro* results indicated the alkaline phosphatase (ALP) activity of the MG-63 cells cultured on the SBA-15/PLGA composite scaffolds showed two-fold higher activity when compared to the pure PLGA scaffolds. Additionally, gene expression studies confirmed that the expression levels of collagen I were significantly higher after 7 days in cells cultured on the SBA-15/PLGA composite scaffold when compared with those cultured on PLGA scaffold [93]. It is worth noting that the pore surface of MSNs can be easily decorated to display a variety of terminal functional groups (amino [94], hydroxyl [95], carboxyl [96] and thiol [97]) using the well-established silane chemistry. The surface modification process results in enhanced physicochemical properties of particles such as those related to sensitivity towards pH, external stimuli, temperature, enzymes, and light. These interventions modulate biocompatibility, drug loading capacity, and targeted drug release [98]. In another study, Szewczyk et al. (2020) loaded cefazolin (Cef) onto amine-functionalised mesoporous silica SBA-15 (SBA-NH_2_-Cef) using the sol–gel method. They separately obtained HAp obtained by microwave-assisted wet precipitation, later combined with SBA-NH_2_-Cef and their excipients (microcrystalline cellulose, ethyl cellulose and polydimethylsiloxane) into a pellet. The obtained pellet exhibited sustained release of Cef for 5 days without any toxic effects. It also displayed beneficial bactericidal effect and induced mineralization upon interaction with human osteoblast. Such combinations of mesoporous silica/polymer composite are ideal for use as drug eluting scaffolds for bone regeneration applications [99]. Lewandowska-Łańcucka et al. (2019) also synthesised amine-functionalised silica particles, which were dispersed into a polymeric solution containing chitosan, collagen, hyaluronic acid solution that was later crosslinked with genipin. The results demonstrated that the composite facilitated bone cell attachment, proliferation, as well as regulation of bone differentiation factors such as alkaline phosphatase (ALP). The bioactive nature of the matrix proves useful for bone regeneration [100].

The targeted release of drug molecules is highly beneficial for tissue healing. MSNs can be employed as an intermediate delivery vehicle where they encapsulate and release the drug molecule themselves. For example, Xu et al. (2020) nanoengineered core-shell composite nanofiber membranes with growth factor and antibiotic delivery capabilities to achieve dual functions. In the core-shell composite nanofibers, the core consisted of PVA incorporated with growth factor recombinant bone morphology protein (rhBMP-2) loaded MSNs, while the shell was fabricated with antibiotic-loaded PCL and spun into nanofibers using coaxial electrospinning. A sustained release behaviour of rhBMP-2 was observed and the drug-loaded nanocomposite core–shell nanofibers showed excellent antibacterial properties toward gram-positive (*Staphylococcus aureus*), gram-negative (*Escherichia coli*), and multispecies oral bacteria as represented in Fig. 4b [84].

### 4.3 MoF/polymer composites

Metal organic frameworks (MoF), also known as porous coordination polymers (PCPs) are synthetic materials that have appeared in various applications for tissue engineering. MoFs consist of organic ligands named as ‘struts’ or ‘linkers’, which are bonded to metal cations or clusters of cations called nodes, all of which give them a crystalline structure [101]. Because of their highly precise and tuneable pore cavities, ordered porous structure, and controllable aperture/sizes. MoFs have become an ideal candidates for various aspects of biomedical engineering such as those related to sustainable drug [102], diagnosis [103] and TERM applications [104,105]. In a study, Xue et al. (2021) electrospun polycaprolactone and collagen (PCL/Col) membrane with MoF modified asymmetric double-layer to trigger a pH-sensitive release of Zn^2+^ ions. This membrane acted as a barrier to prevent fibrous connective tissue infiltration in guided bone regeneration. The use of MoF crystals inherently induced both osteogenesis and angiogenesis after 8 weeks of transplantation in calvaria defect model (Fig. 4c). Similar observations of enhanced angiogenic response were also reported in chick chorioallantois membrane, making it a promising material for bone regeneration [85].

Darder et al. (2020) developed biohybrid structures by assembling cellulose micro and nanofibers with copper-cystine (CuHARS) and then coated with polyallylamine hydrochloride (PAH). The Cu^2+^ ions present in the composite acted as a catalyst to produce nitric oxide (NO) from available bioresources. The NO-releasing composite prevented microbial infection, thereby reducing bacterial adhesion and colonization. Therefore, such NO releasing MoF-based composites can become an active ingredient for the next generation of wound dressings [106]. In another study, Ramezani et al. (2019) developed nanofibrous polycaprolactone (PCL) by electrospinning, which embedded a Fe ion based MoF synthesised via a hydrothermal method. The final composites were highly porous in nature and biocompatible. The biodegradation behaviour of this composite was highly promising both *in vivo* and *in vitro*. The Fe-MoF composites also supported attachment of human umbilical vein endothelial cells (HUVECs) and promoted viability. These results demonstrated that the PCL-Fe-MoF composites can be useful in epidermis regeneration related applications [107].

## 5 Miscellaneous inorganic materials/polymer composites

Besides the solid and porous inorganic particles described in the sections above, a wide variety of other inorganic materials such as metal ions [108], nanofiber [109], nanosheets, and nanoclays [110] have been incorporated into polymers for the creation of inorganic-organic polymer combination composites. These new inorganic materials are rapidly becoming popular due to their promising properties including chemical inertness, low thermal conduction, and low cytotoxicity [[111], [112], [113], [114]]. In the recent years, advanced inorganic-organic combination composites based on these inorganic materials have been widely used in many biomedical applications, including tissue engineering [[115], [116], [117], [118]].

### 5.1 Metal particles and ions incorporated polymer composites

**Fig. 5:**
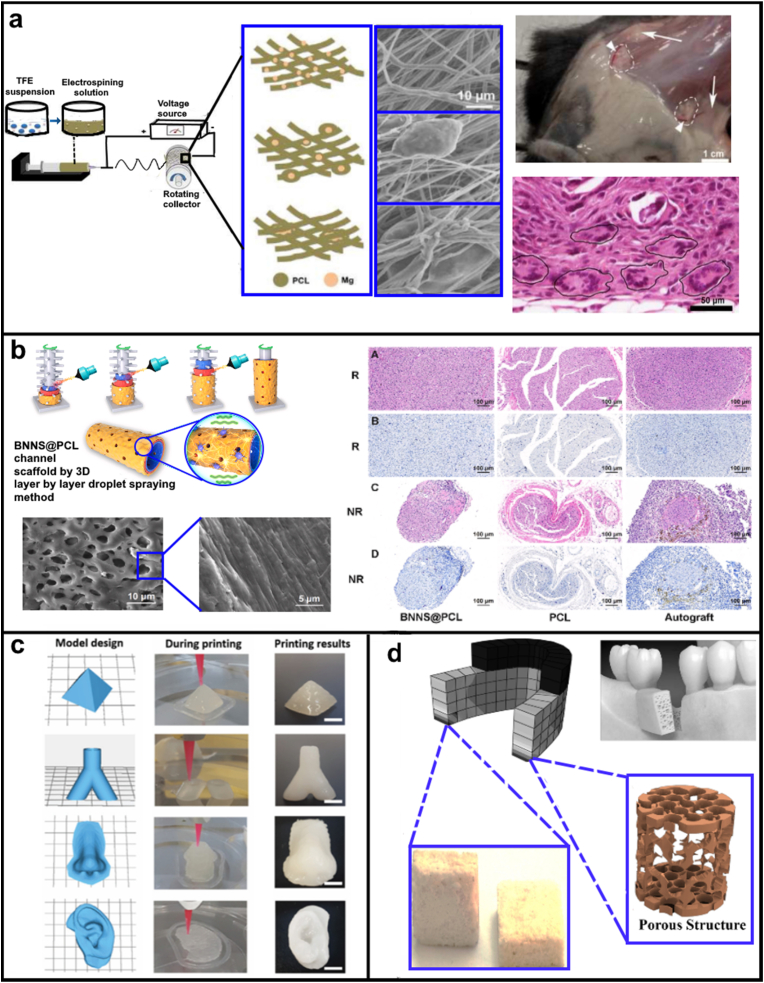
(a) Schematic representation of PCL-Mg composite nanofiber mesh preparation. Mg particles enmeshed within the PCL nanofibers as depicted in the drawings and SEM images. *In vivo* tissues after 28 DIV showed large blood vessels (white arrowheads) and large lateral fat pads (white arrows). H&E-stained sections showed that mesh interior contained numerous macrophages and multinucleated FBGCs (black circles). Reproduced from Ref. [124] (b) A piezoelectric scaffold is fabricated by 3D layer-by-layer droplet spraying method and favors microenvironment rebalance cocktail therapy. Histology showing sciatic nerve morphology and myelinated fiber observed in transmission electron microscope following the application boron nitride nanosheets for piezocatalytic neuronal repair. Reproduced from Ref. [128]. (c) 3D Printing of nanoclay hydrogels into complex architecture which can use in regeneration of complex defects. Reproduced from Ref. [129]. (d) Bioactive nanoclay-TiO_2_ (NC-T) scaffolds containing TiO_2_ fractions fabricated with spacer for bone tissue engineering via the space holder technique using NaCl particles. The microstructure and surface morphology showed porosity and potential for manufactured bio-nanocomposite scaffolds. Reproduced from Ref. [130].

Recently, metal particles and ions-based polymer composites have emerged as an important material for biomedical applications due to their small diameters, large surface area per unit volume, and ease of functionalisation all of which improve their receptor binding capabilities [119]. These are commonly applied to a broad number of tissue engineering [120], drug delivery [121], cancer therapy [122] and regenerative medicine interventions [123]. For example, Adhikari et al. (2019) incorporated magnesium particles into polymeric nanofibers using electrospinning that simulated the tissue repair by increasing the activity and infiltration of macrophages through collagen matrix deposition and organization (Fig. 5a) [124]. In a similar study Jaidev et al. (2017), synthesised copper particles decorated onto graphene oxide and dispersed in polycaprolactone matrix to enhance regeneration of bone tissue. The multifunctional composite was aimed at promoting better osteogenic and angiogenic functions, while eliciting bactericidal effect at the same time to prevent infection during the initial part of the healing [125]. Anamizu et al. (2019) designed injectable hydrogels by mixing FeCl_3_ solution and alginate/gelatin solution at various ratios (A10G00, A08G02, A07G03, A05G05, A03G07, A02G08, and A00G10 with wt% of 10:0, 8:2, 7:3, 5:5, 3:7, 2:8, and 0:10, respectively). The dissolution rate of ferric ions from hydrogels in phosphate buffered-saline solution (PBS) containing collagenase increased with the decrease in alginate/gelatin ratio. This was attributed to the ease of gelatin degradation by collagenase and strong interaction of ferric ions with alginate as compared to gelatin. The viability and proliferation of MC3T3-E1 cells (murine bone calvaria pre-osteoblast) increased with a decrease in alginate/gelatin in the hydrogel. For *in vivo* cell transplantation, cells encapsulated with A2G8 hydrogel were implanted into the back subcutis of C57BL/6n mouse. The percentages of cells retained after the PBS injection (control) and cells encapsulated in the A2G8 hydrogel were 3.27 and 82.5% respectively for day 1 and 0.18% and 32.8% respectively for day 3. It can be concluded that this metal ions-based injectable hydrogel proved promising for cell transplantation therapy. There are other examples of metal ions used in conjunction with alginate to create biomaterials and scaffolds for tissue engineering, which are covered in detail in other recent reviews [126,127].

### 5.2 Inorganic nanosheet-based polymer composites

Nanosheet are planar two-dimensional (2D) structures with monolayer or multilayered stacking arrangement. Typically, 2D inorganic nanosheets possess higher mechanical strength and an enormous surface area to volume ratio [131]. Graphene is a key example of 2D nanosheet like material that has been applied in various applications in tissue engineering. However, it is out of the scope of this review due to its hydrocarbon based back-bone. New inorganic nanosheet like 2D materials have been developed and refined in the last two decade including molybdenum disulphide, boron nitride nanosheets, black phosphorus nanosheets for biomedical applications including tissue engineering [132,133]. In this direction, Yun et al. (2021) synthesised piezoelectric boron nitride nanosheets (BNNS) incorporated porous PCL scaffold using an interesting 3D layer-by-layer droplet spray method. In this process, porous scaffolds were generated by spraying PCL or BNNS functionalised PCL onto a rotating drum with microneedles projections. The smart BNNS@PCL porous scaffold induces micro-vessel regrowth into neurons and reverses muscular atrophy after denervation in a severe sciatic nerve defect model *in vivo*. As represented in Fig. 5, 3D nano-scaffold improved muscle reinnervation and locomotor recovery. Thus, BNNS functionalised interface can be a promising alternative for nerve tissue engineering and can possess potential for clinical translation [128].

### 5.3 Inorganic nanoclays based polymer composite

Nanoclays are natural minerals with the diameter size range of 1–100 nm. Natural nanoclays exist in two forms, as either anionic or cationic clays depending upon the charge of their surface layer and the type of interlayer ions present [134]. In the last decades, synthetic nanoclay materials have been developed to overcome the variability and purity issues of the natural nanoclays. The tuneable size, shapes, and biocompatibility of nanoclay make them particularly versatile and beneficial for wound healing [135], tissue engineering [136], cancer therapy [137], drug delivery [138] and enzyme immobilization [139] applications. Nanoclays can be modified to develop self-supporting, self-recovery, and extrusion based 3D printable nanoclay-incorporating double-network (NIDN) hydrogel biomaterial ink to form mechanically strong 1D filaments and 3D constructs [129]. Hydrogel biomaterial ink was comprised of nanoclay (Laponite XLG), methacrylate hyaluronic acid (HAMA), and alginate. Various 3D constructs geometries such as pyramid, human nose, vascular and human ear were effectively printed with this bio-ink (Fig. 5c) [129]. Nanoclays have been extensively used in polymer composites as reinforcement material to enhance thermal, mechanical, and anticorrosion characteristics [140]. For example, Yao et al. (2020) developed 3D gelatin nanofibrous scaffold (GF/NS) functionalised with nanoclay-nanosilicate (NS). The composite scaffolds (GF/NS) significantly increased mechanical strength, and promoted osteogenic differentiation of human mesenchymal stem cells (hMSCs) [141]. Zheng et al. (2021) developed a nanoclay (Laponite, XLS) functionalised 3D bioglass loaded GelMA-Desferoxamine (GelMA-DFO). The composite facilitated the sustained release of DFO and induced vascular endothelial growth factor (VEGF) expressions in human adipose mesenchymal stem cells (ADSCs), which altogether promoted angiogenesis and osteogenic differentiation of the stem cells for endogenous bone repair [142].

Other intervening strategies are aimed at directly altering the mechanical properties of the final nanocomposites. Sahmani et al. (2018) used this approach to create a nanoclay composite of chitosan containing PVA and Montmorillonite as represented in Fig. 5d. The resuspension of the PVA and Montmorillonite mixture into chitosan solution yields a greater than 30% increase in tensile strength when compared to non-functionalised hydrogel counterpart, upon free drying. The nanocomposite exhibited good swelling behaviour and bactericidal effect which are desirable properties for wound dressing [130].

## 6 Conclusion

Tissue engineering and regenerative medicine (TERM) is undergoing rapid evolution, moulded by fast paced advances in several interdisciplinary fields including biomaterials, bioengineering, additive manufacturing, and advanced manufacturing. Biomaterials research has had the most significant impact on TERM through increasing our understanding of natural tissue and utilising this knowledge for designing new biomaterials that mimic these properties. New biomaterials discoveries have already entered clinical use in various subfields of TERM including bone, skin substitutes, cornea, nervous system, cardiovascular, and dental regenerations. Of the various materials, composites are the materials of choice for many TERM applications. This review emphasises on inorganic/organic combination composites made by incorporating inorganic particles into polymeric matrices. Such composites are of particular use because there is a huge variety of inorganic materials with a gambit of interesting physico-chemical and biological properties desirable for TERM applications. Tremendous progress has happened in generation of inorganic/organic combination composites with studies showcasing use of solid as well as porous inorganic particles in polymer matrix. Solid particles of both metallic (e.g., gold, and silver nanoparticles) and non-metallic origin (e.g., calcium phosphate and hydroxyapatite particles) have been incorporated into polymers to generate composite scaffolds. Primary purpose of incorporating solid particles in the composites, in majority of studies, has been to improve cellular interaction for an enhanced tissue regeneration. While silver nanoparticles in a scaffold often serve dual purpose of enhanced cellular interaction as well as anti-bacterial activity. Most porous nanoparticles (mesoporous silica, porous silicon, metal organic framework, *etc.*) offer intriguing features such as high surface area, ability to incorporate and deliver payloads (small molecules and macromolecular therapeutics), while porous silicon is inherently photoluminescent. All these properties have resulted in a plethora of investigations incorporating inorganic porous particles in polymer composites for TERM applications.

Although tremendous amount of research has been undertaken on generation of inorganic particle/polymer composite scaffolds, their clinical translation has been impacted by a long list of unknowns. Though many of the reviewed inorganic materials have been investigated for their short-term safety both in cellular and animal models, our knowledge of the long-term safety of the inorganic materials and the ionic species eluted upon their bio-resorption lacks significantly. For the interesting inorganic/organic combinations scaffolds summarised in this review to translate from lab to clinic, they will need to go through a long regimen of safety assessment that matches the intended operational life of the scaffold. The short-term safety and efficacy studies, as they are reported currently, are due to lack of animal models that mimic the human disease as well as extremely high cost of conducting long-term safety studies. In addition, despite the progress, there is very limited understanding of impact of morphology and mechanical properties of inorganic particles on cellular functions both *in vitro* and *in vivo.* There is also a huge scope for utilising porous nanocarriers based scaffolds for controlled localised delivery of biological therapeutic payloads that has been poorly investigated so far. Lastly, one of the biggest challenge plaguing lab-to-clinic transition of inorganic/organic combination composite scaffolds is the lack of relevant *in vitro* and *in vivo* models. Therefore, more concentrated efforts are needed in design and development of new *in vitro* models such as organ-on-chip devices to study efficacy and safety of composite tissue engineering scaffolds. In future, we envision, inorganic/organic combination composite will lead to smart tissue engineering scaffolds with ability to delivery therapeutic payloads at site, modulate inflammatory, cytokine, and cellular microenvironment around the graft site, guide cellular and/or cell structure growth in a desired manner, and report scaffold health remotely, while also being safer in the long-term.

## Ethics approval and consent to participate

This review article does not include any original animal or human research data. Wherever necessary, we have obtained the copyright permission for reproduction of the figures and tables in the review.

## CRediT authorship contribution statement

**Astha Sharma:** Formal analysis, Writing – original draft, Writing – review & editing, carried out the literature search and analysis, manuscript drafting, figure preparations, and editing. **Ganesh R. Kokil:** Formal analysis, Writing – original draft, Writing – review & editing, carried out the literature search and analysis, manuscript drafting, figure preparations, and editing. **Yan He:** Formal analysis, Writing – original draft, Writing – review & editing, carried out the literature search and analysis, manuscript drafting, figure preparations, and editing. **Baboucarr Lowe:** Writing – review & editing, assisted in reviewing and editing the manuscript. **Arwa Salam:** Writing – review & editing, assisted in reviewing and editing the manuscript. **Tariq A. Altalhi:** Writing – review & editing, assisted in reviewing and editing the manuscript. **Qingsong Ye:** Conceptualization, Supervision, Writing – review & editing, Conceptualised the review and supervised the team, and also played key role in review & editing of the manuscript. **Tushar Kumeria:** Conceptualization, Supervision, Writing – review & editing, Conceptualised the review and supervised the team, and also played key role in review & editing of the manuscript.,.

## Declaration of competing interest

The authors declare that they have no known competing interests that could have influenced the literature reported in this paper.
